# Dimensional latent structure of internet gaming disorder symptoms in four representative surveys of German adolescents: results from taxometric analyses

**DOI:** 10.3389/fpsyt.2025.1606793

**Published:** 2025-09-15

**Authors:** Sören Kliem, Sebastian Fischer, Yvonne Krieg, Dirk Baier, Florian Rehbein

**Affiliations:** ^1^ Ernst-Abbe-Hochschule Jena – University of Applied Sciences, Jena, Germany; ^2^ Institute of Delinquency and Crime Prevention, Zurich University of Applied Sciences, Zurich, Germany; ^3^ University of Zurich, Zürich, Switzerland; ^4^ FH Münster, University of Applied Sciences, Münster, Germany

**Keywords:** internet gaming disorder, taxometrics, latent structure, behavioral addictions, psychometric assessment

## Abstract

**Background:**

A very large amount of research has addressed the issue of the latent status of psychiatric disorders. To our knowledge, no study has analyzed the latent structure of Internet Gaming Disorder (IGD) symptoms.

**Method:**

We used a new taxometric approach developed by Ruscio et al. rather than estimating a putative taxon base rate and using that estimate to generate the taxon comparative data, we estimate CCFI-profiles with each base rate estimate between.025 and.975 in increments of.025. Nine indicators (1. Preoccupation, 2. Withdrawal, 3. Tolerance, 4. Reduce/stop, 5. Continue despite problems, 6. Give up other activities, 7. Escape adverse moods, 8. Deceive/cover up, and 9. Risk/lose) according to the prescriptions of the DSM-5 were used as well as a four-indicator set based on ICD-11. The analyses draw on data from German ninth-grade students collected between 2013 and 2019 as part of a periodic representative survey. Overall, *N* = 36 630 (response rates: 41.4-68.5%; 50.2% male, 27.3% with migration background) adolescents were reached. The Video Game Dependency Scale (CSAS) was used to assess IGD symptoms in accordance with DSM-5.

**Results:**

Regarding the total sample (DSM-5: CCFI-mean-profile = 0.311; ICD-11: CCFI-mean-profile = 0.175), the male sample (CCFI-mean-profile = 0.162/0.046), and female sample (CCFI-mean-profile = 0.390/0.268), strong support for the superiority of a dimensional model was detected.

**Conclusion:**

It seems necessary to define diagnostic thresholds regarding IGD-symptom burden based on external criteria (e.g., IGD-related incapacity to work or truancy). Further studies are necessary to substantiate this result in different samples using different measurement approaches.

## Introduction

Behavioral addictions are characterized by the compulsive engagement in activities that initially offer rewarding experiences, but over time lead to clinically significant impairments and negative long-term consequences (1, 2). With the introduction of the fifth edition of the Diagnostic and Statistical Manual of Mental Disorders (DSM-5; 3), the concept of behavioral addiction was formalized, and Gambling Disorder was classified alongside substance use disorders as the first officially recognized non-substance-related addiction. In the same context, Internet Gaming Disorder (IGD) was included in Section III of the DSM-5 as a condition warranting further clinical and empirical investigation (4). IGD is defined as a maladaptive pattern of persistent (online) gaming behavior, resulting in functional impairments across personal, social, educational, or occupational domains. The DSM-5 (3) lists nine diagnostic criteria, of which at least five must be met within a 12-month period for a tentative diagnosis. These criteria, adapted from substance use and gambling disorder frameworks, cover a range of behavioral, emotional, and cognitive symptoms:

### Preoccupation with gaming (#1)

Individuals experience intrusive thoughts about gaming, even when not actively engaged in it. This includes rumination about past gaming sessions, fantasizing about future gameplay, and prioritizing gaming over other cognitive content. This criterion reflects the construct of *cognitive salience*, a hallmark of behavioral addiction.

### Withdrawal symptoms (#2)

Emotional or physical discomfort (e.g., irritability, anxiety, sadness) when gaming is reduced or terminated. These reactions are not merely frustration at being interrupted but resemble withdrawal states known from substance-related disorders. Symptoms must occur in the absence of the behavior and not only as a situational response (e.g., being interrupted during play).

### Tolerance (#3)

A marked increase in time or intensity required to achieve the same level of satisfaction. This may manifest as longer gaming sessions, engagement with more stimulating or competitive games, or the use of enhanced in-game equipment or environments. Tolerance implies neurobehavioral adaptation over time.

### Unsuccessful attempts to reduce or stop gaming (#4)

Repeated efforts to cut down on gaming are unsuccessful. This criterion requires the presence of insight into problematic use and the intention to change, without successful behavioral implementation. It reflects impaired self-regulation and control over the activity.

### Loss of interest in other activities (#5)

Reduction or abandonment of previously valued hobbies, social activities, or recreational pursuits. This behavioral narrowing signals that gaming has become the dominant activity, often at the expense of interpersonal or academic functioning (*behavioral salience*).

### Continued use despite negative consequences (#6)

Persistence of gaming behavior despite awareness of its harmful effects (e.g., fatigue, declining academic performance, interpersonal conflict). This reflects impaired judgment and motivational salience of gaming, overriding external contingencies.

### Deception or concealment of gaming behavior (#7)

The individual lies to family members, therapists, or others about the amount of time spent gaming, or conceals the behavior entirely. Deception is typically directed toward preserving access to gaming and minimizing external criticism.

### Gaming to deceive or relieve negative mood states (#8)

Gaming is used as a coping mechanism for stress, guilt, anxiety, or depressive mood. This is distinguished from gaming to avoid withdrawal (criterion 2); here, gaming serves primarily to modulate emotional distress originating outside the gaming context.

### Jeopardizing or losing a relationship or opportunity due to gaming (#9)

Serious adverse consequences occur, such as the loss of a close relationship, job, or educational opportunity. The threshold for this criterion is higher than for criterion 6 and requires evidence of *clinically significant* impairments or losses.

In addition to its inclusion in the DSM-5 as a condition warranting further study, Internet Gaming Disorder (IGD) was also incorporated into the revised version of the International Classification of Diseases (ICD-11). The ICD-11 formally recognized Gaming Disorder as a behavioral addiction in 2019 (5). The ICD-11 includes a more concise diagnostic framework, consisting of three symptom criteria - impaired control, increased priority, and continued use despite harm - which correspond closely to DSM-5 criteria #4, #5, and #6. An additional impairment criterion (akin to DSM-5 criterion #9) must also be present. Notably, all four ICD-11 criteria must be fulfilled within a 12-month period to assign a diagnosis.

While both diagnostic systems adopt a categorical classification model, distinguishing between “disordered” and “non-disordered” individuals based on threshold criteria, recent conceptual and empirical developments challenge this binary view. An alternative and increasingly supported approach is the conceptualization of IGD as the end of a continuous distribution of gaming-related symptoms (6, 7). Such a dimensional perspective reflects the growing body of literature in psychopathology that regards many mental disorders as quantitative phenomena, rather than discrete clinical entities (8). Although the latent structure of a construct or condition, does not decide upon its existence, it is an important issue for multiple reasons implication for research, theory, and practice (9, 10): First, the latent status of a construct is important for the classification of individuals. If the underlying construct is continuous, the convention for classification into dichotomous groups (diseased vs. healthy) must be derived based on certain criteria that are not part of the diagnosis (external validation criteria). On the other hand, if a true categorical latent structure exists, providing clinically relevant cut-off values to differentiate the respective groups appears to be an important target. Second, the latent status of a phenomenon is important for developing assessment tools. In the case of a categorical latent structure, it seemed reasonable to focus on items that discriminate at most between groups. On the other hand, if the construct is continuous, items across the entire spectrum of the latent continuum should have to be included to be able to discriminate over the entire spectrum of the phenomenon. Third, in the context of evaluation studies, the latent status of a phenomenon appears to be of particular importance and should also affect the selection of the appropriate evaluation criterion. Thus, in the context of a dimensional construct, the use of effect sizes (ES) seems appropriate (i.e., change in the severity of expression on the latent continuum), whereas the calculation of clinical significances (11, 12) represent a categorical conception (i.e., change of the category ‘ill’ to ‘healthy’). Lastly, information about latent status can provide eminent theoretical insights. For example, Meehl (13) argues that a dimensional structure can be reached as a result of a multitude of minor risk factors that act via addition and interaction. On the other hand, existence of categorical latent structure can result from a specific etiology or developmental bifurcation. In the context of emerging disorders such as Internet Gaming Disorder (IGD), where nosological status and underlying structure remain unsettled, it is essential to empirically evaluate the latent nature of the construct rather than assume it *a priori*.

An extensive body of research has addressed the issues of the latent status of psychiatric disorders (14–18). Taxometric methods, originally developed by Meehl (13) and further refined by Ruscio and colleagues (19), are explicitly designed for this purpose. Importantly, taxometric analysis does not rely on arbitrary model fit indices or class enumeration criteria, as is common in latent class analysis (LCA) or latent profile analysis (LPA), which often presuppose categorical solutions. A central advancement in modern taxometric practice is the Comparison Curve Fit Index (CCFI), introduced by Ruscio et al., which quantitatively compares the fit of empirical data to simulated taxonic and dimensional datasets (9). To increase interpretive reliability and reduce dependence on single index values, Ruscio et al. introduced CCFI profile analysis (20). This approach examines CCFI values across multiple valid indicator combinations and base rate estimates, thereby creating a profile of CCFI values that provides a more stable and nuanced assessment of latent structure. Unlike model-based approaches such as factor analysis or mixture models, which may produce plausible solutions regardless of the true latent structure, taxometric procedures are specifically calibrated to adjudicate between dimensional and taxonic nature.

With regard to the latent structure of behavioral addictions, there are so far only a few findings on problem gambling (PG) which provide inconsistent findings regarding the latent structure of the phenomenon. James et al. (21) for example examined problem gambling using two problem gambling screens within the British Gambling Prevalence Survey. There was strong evidence that both scales measure a categorical construct. Furthermore, Kincaid et al. (22) investigated problem gambling (PG) in a 2010 South African sample (*N* = 3,000). Again, results indicate positive but modest evidence for a categorical structure. On the other hand, Braverman et al. (23) failed to provide support for a distinct category of PG. To the best of our knowledge, no previous study has analyzed the latent structure of IGD-symptoms. Thus, the primary objective of the present study was to examine the latent structure of IGD symptoms among adolescents using latest taxometric methods (CCFI profile), in order to determine whether IGD constitutes a categorical disorder or reflects a dimensional construct along a continuum of severity. Although IGD has been provisionally included in the DSM-5 and ICD-11, the empirical basis regarding its latent structure remains limited. This is noteworthy given the increasing prevalence and psychosocial impact of IGD worldwide. Recent meta-analytic data indicate that approximately 8.6% of adolescents meet the diagnostic criteria for Gaming Disorder, with evidence suggesting an upward trend over the past decade (24). Notably, the COVID-19 pandemic appears to have further intensified problematic gaming behaviors. Lockdowns, school closures, and social distancing measures have increased screen time among young people, often replacing offline social interaction and structured daily routines. Several studies report a post-pandemic rise in IGD symptomatology, along with associated impairments in academic performance, sleep, and mental health (25, 26). These findings underscore the clinical and public health significance of IGD, especially in adolescent populations. Moreover, diagnostic systems differ in how IGD is conceptualized: while the DSM-5 defines IGD as a condition for further study and outlines nine behavioral criteria, the ICD-11 conceptualizes Gaming Disorder as a formal mental disorder with a stricter threshold and fewer criteria. These structural and definitional differences raise the important question of whether the symptomatology implied by each system reflects the same underlying latent construct. It would be diagnostically problematic if, for example, IGD based on DSM-5 criteria follows a dimensional structure, while ICD-11-based criteria yield evidence for a categorical disorder.

## Materials and methods

### Participants and procedure

The following analyses draw on data from German ninth-grade students collected in 2013, 2015, 2017, and 2019 as part of a periodic representative [see (27–29)]. The present analyses draw on data from German ninth-grade students collected in 2013, 2015, 2017, and 2019 as part of a periodic, representative school survey. The highest priority across all four survey waves was to design a study that would yield representative data for the state of Lower Saxony. This was made possible through a school-class-based survey design. The term “school-class-based” implies that a random selection of school classes was drawn from a comprehensive list that included all ninth-grade classes in Lower Saxony and was provided by the Statistical Office of Lower Saxony. All school types were included in the sampling process, with the exception of special-needs schools focusing on areas other than learning. The exclusion of special-needs schools for students with, for example, intellectual or physical disabilities is due to the fact that survey-based data collection via questionnaire is not feasible in these settings. In each wave, sampling was conducted at the classroom level and stratified by school type. Permissions to conduct the study in schools were obtained in accordance with the directive issued by the Lower Saxony Ministry of Education (RdErl. d. MK v. 1.1.2014–25b–81402 – VORIS 22410) via the State School Authority of Hanover. The proportional selection of schools was based on the distribution of school types in the population; however, no regional stratification was applied. The federal school board of Lower Saxony as well as the Ministry of Education of Lower Saxony (which constitutes the state’s educational authority) approved the survey and provided ethics auditing. Furthermore, the “Niedersachsensurvey” was approved by the Ethics Committee of the University of Göttingen (08122023). All procedures involving human participants were carried out in accordance with institutional and national ethical standards. The survey was strictly anonymized – neither names, nor private or school addresses were obtained. The students’ parents received an information leaflet beforehand, which included a request for written consent for the participation of their child and provided them with information about aims, methods and funding of the study. Students were informed that participation in the survey was entirely voluntary and anonymous and that they could withdraw from participating at any time and without any negative consequences. Furthermore, they were informed of their right to skip individual questions within the survey and were encouraged to speak to a counsellor or school psychologist should participation in the survey have affected them negatively in any way. In 2013, a total of *N* = 9,512 adolescents from *K* = 485 classes participated in the survey, corresponding to a response rate of 64.4% (50.7% male; 24.3% with a migration background). In 2015, *N* = 10,638 adolescents from *K* =545 classes were surveyed (response rate: 68.5%; 50.1% male; 24.0% with a migration background). In 2017, the survey reached *N* = 8,938 adolescents from *K* = 479 classes (response rate: 59.2%; 49.0% male; 27.7% with a migration background). In 2019 a total of *N* = 12,444 adolescents from *K* = 762 classes were (response rate: 41.4%; 50.9% male, 31.1% with a migration background) were reached. Due to the modular structure of the 2019 questionnaire, the items assessing Internet Gaming Disorder (IGD) were presented to only about one quarter of the adolescents. Overall, *N* = 36,630 (50.2% male, 27.3% with migration background) adolescents were analyzed. The main reasons for non-participation were missing parental consent (*n* = 2,765, based on data from the 2013, 2015, 2017, and 2019 survey waves), illness (*n* = 766), and lack of adolescent assent (*n* = 683). In addition, *n =* 192 questionnaires were classified as invalid, *n* = 33 students were absent due to truancy, and *n* = 1,103 cases were excluded for other or non-reconstructable reasons. A detailed sample overview can be found in **Table 1**.

**Table 1:** Demographic characteristics of the pooled study sample.

Sample characteristics	Male (N = 18 406)	Female (N = 18 224)	Total (N = 36 630)
Age			
13	61 (0.3%)	38 (0.2%)	99 (0.3%)
14	4,414 (24.0%)	5,815 (31.9%)	10,229 (27.9%)
15	10,232 (55.6%)	9,941 (54.5%)	20,173 (55.1%)
16	3,167 (17.2%)	2,087 (11.5%)	5,254 (14.3%)
17	488 (2.7%)	311 (1.7%)	799 (2.2%)
18	44 (0.2%)	32 (0.2%)	76 (0.2%)
Migration Background			
no	13,593 (73.9%)	12,044 (71.6%)	26,637 (72.7%)
yes	3,813 (26.1%)	5,180 (28.4)	9,993 (27.3%)
School type			
low	2,660 (14.5%)	2,302 (12.6%)	4,962 (13.5%)
medium	5867 (31,9%)	5,570 (30.6%)	11,437 (31.2%)
high	9,879 (53.7%)	10,352 (56.8%)	20,231 (55,2%)

### Measures

The Video Game Dependency Scale (CSAS) (30) was used to assess IGD symptoms in accordance with DSM-5. Each DSM-5 criterion was represented by two items, resulting in an 18-item scale. Participants were instructed to respond based on their gaming behavior within the past 12 months and to rate each item on a four-point scale: 1 = disagree at all, 2 = disagree somewhat, 3 = agree somewhat, 4 = agree fully. Although the CSAS was originally developed based on the DSM-5 criteria for IGD, several of its items also show strong conceptual overlap with the four core diagnostic features of Gaming Disorder as defined by the ICD-11 and can therefore be reasonably used for analyses based on the ICD-11 framework as well. The CSAS has further demonstrated strong psychometric properties in large-scale German adolescent samples, including high internal consistency (Cronbach’s α >.85), robust factorial validity, and meaningful associations with external criteria such as academic functioning and psychosocial impairment (30). Alternative measures such as the Internet Gaming Disorder Scale – Short Form (IGDS9-SF) (31) or the Gaming Disorder Test (GDT) (32) which are increasingly used in international contexts, were not validated in German youth samples at the time of data collection.

### Statistical analysis

#### Missing data

To account for missing data, we applied chained equation modeling using the following variables: gender, age, migration background, school type (low, medium, high), time of measurement, and computer play duration per day to estimate missing data. To avoid implausible item values, the estimated values (y) were corrected by predictive mean matching (i.e., the observable values closest to the predicted value were chosen). We used the R package *mice* (33) for imputation.

#### Indicator selection

We used all 18 items of the CSAS with the sum score of two items forming one indicator (sum score: min= 2 to max = 8). We analyzed two different indicator sets including nine indicators according to the prescriptions of the DSM-5 and four indicators according to the prescriptions of the ICD-11 (see **Table 2**).

**Table 2:** Characteristics of the included indicators.

Criteria/Indicators	Descriptive Statistics				Cohen’s d	
	M	SD	Skewness	Kurtosis	DSM-5 indicator set	ICD-11 indicator set
Preoccupation,	0.89	1.30	1.61	2.20	2.36	–
Withdrawal,	0.47	1.03	2.62	7,38	2.96	–
Tolerance,	0.75	1.22	1.80	3.09	2.89	–
Reduce/stop,	0.56	1.06	2.26	5.42	2.78	3.63
Continue despite problems,	0.41	0.97	2.92	9.36	2.99	4.36
Give up other activities,	0.53	1.05	2.32	5.61	3.10	3.79
Escape adverse moods,	0.53	1.12	2.49	6.33	2.78	–
Deceive/cover up	0.92	1.52	1.68	1.93	2.32	–
Risk/lose	0.31	0.84	3.40	13.42	2.76	4.76

#### Taxometric analysis

As recommended by Ruscio et al. (34), we applied three non-redundant taxometric procedures:

Mean above minus below a cut [MAMBAC (35)], maximum eigenvalue [MAXEIG (36)], and latent-mode factor analysis [L-Mode (36)]. All three procedures can be interpreted graphically: if the graphical output will yield a peaking curve for the MAMBAC and MAXEIG analyses and a multi-modal distribution curve for the L-Mode procedure, a categorical structure is present (31). Following the suggestion by Ruscio et al. (9, 37), two comparison populations (each N = 100,000) using (a) the categorical model and (b) the dimensional model, were generated for each of the taxometric procedures. Relevant aspects of the empirical data, such as skewness, e.g., inter-correlations, and non-normality, were held constant. In a second step, random samples (K= 100; with the same sample size of the empirical data set) were drawn from both populations. All samples were then analyzed using the three different taxometric procedures (MAMBAC, MAXEIG, L- MODE). In addition to the graphical output, the root-mean-square distance between empirical data points on curves and data points on simulated categorical (FitCat) as well as simulated dimensional (FitDim) reference curves were calculated (smaller values indicating that both curves more closely resemble one another). Next, the comparison curve fit index (CCFI = FitDim/(FitDim + FitCat)) was calculated for each taxo- metric procedure. A CCFI value above 0.50 denotes a better fit for a categorical latent structure and a value below 0.50 denotes a better fit for a dimensional latent structure. Finally, in accordance with Ruscio et al. (34), the mean CCFI of the MAMBAC, MAXEIG, and L-Mode procedure was used to interpret the latent status of IGD. The accuracy of this criterion is supported by a large simulation study by Ruscio et al. (34) which concluded that using the mean CCFI with a threshold of 0.5 achieved 98% accuracy in correctly classifying the latent status of a construct. Nevertheless, we used a new taxometric approach developed by Ruscio et al. (20), the CCFI profile method. Rather than estimating a putative taxon base rate and using that estimate to generate the taxon comparative data, the CCFI profile method replicates the analysis with each base rate estimate between.025 and.975 in increments of.025. If the construct is taxonic, the CCFI (20) value should be greatest at the most accurate base rate estimation. In Monte Carlo simulations, this method provided a more accurate base rate estimation (in the case of categorical structure) as well as a particularly adequate estimate of latent structure on the basis of a CCFI profile value, whereby a CCFI profile value above 0.50 denotes a better fit for a categorical latent structure and a value below 0.50 denotes a better fit for a dimensional latent structure (20). We used Ruscio’s and Wang’s R package *taxometrics* (38) for the analysis. We performed CCFI profile analysis for the total sample as well as for males and females separately.

### Suitability of data for taxometric analysis

To check the prerequisites for taxometric analysis we used a group variable (taxon vs. complement) based on a DSM-5/ICD-11 algorithm (five of nine and four of four criteria had to be met). Taxometric analysis requires that all standardized mean differences between the hypothetical categorical groups are larger than Cohen’s d = 1.25. Furthermore, all indicators should correlate substantially with each other (mean *r*>0.30), but the correlation should be substantially smaller within the hypothetical categorical groups (*r_wg_* ≤ 0.30) (31).

## Results

### Pre-taxometric analyses

**Table 1** presents the demographic characteristics of the pooled study sample (N = 36,630), stratified by sex. The sample is nearly balanced between male (n = 18,406) and female (n = 18,224) participants. The majority of respondents were aged 14 to 16 years, with 15-year-olds comprising the largest single age group (55.1 % of the total sample). Approximately 27.3 % of the adolescents reported a migration background, with a slightly higher proportion among females (28.4 %) than males (26.1 %). In terms of educational level, the sample includes adolescents from all school tracks, with the majority attending higher-track schools (55.2 %), followed by medium-track (31.2 %) and lower-track schools (13.5 %). **Table 2** summarizes the psychometric characteristics of the indicators used to operationalize the DSM-5 and ICD-11 criteria for Internet Gaming Disorder (IGD). For each symptom domain, the mean (M), standard deviation (SD), skewness, kurtosis, and Cohen’s d effect sizes are reported. Cohen´s d, reflecting contrasts between individuals with low versus high symptom burden, reveal consistently large values across all IGD indicators which substantially exceed the recommended minimum threshold of d = 1.25 for taxometric analyses (34). These large values indicate that the indicators are well suited for distinguishing along a latent severity continuum. We observed an average correlation of *r*=.059/0.61 for the DSM-5 and ICD-11 indicator sets, respectively. A much smaller correlation in the hypothetical categorical groups (DSM-5: *r*=0.23 [taxon], *r*=0.42 [complement]; ICD-11: *r*=0.38 [taxon], *r*=0.51 [complement]) for both indicator sets were detected.

### Taxometric analyses

**Figure 1:**
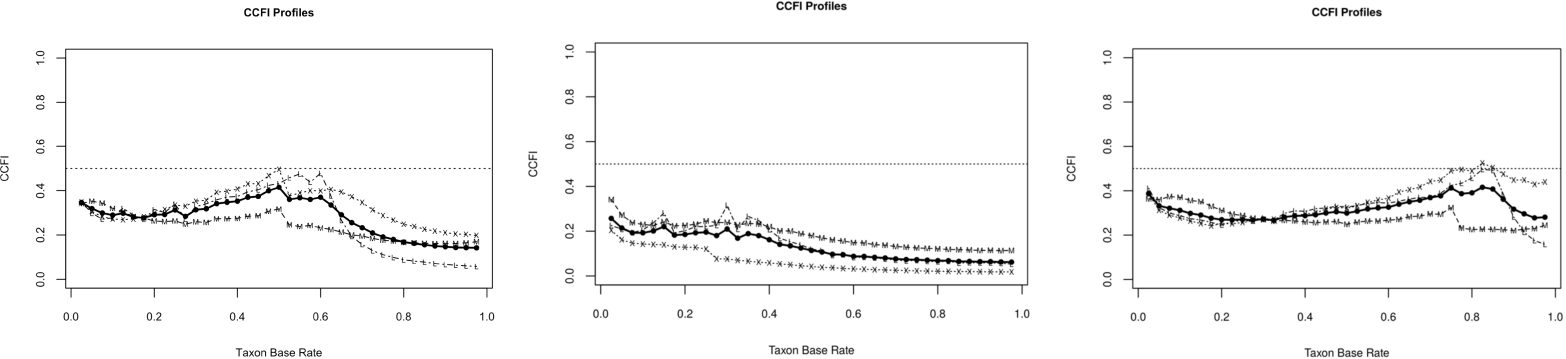
Results of the CCFI-profile analyses based on the DSM-5 indicator-set for the total sample (right), male sample (middle), and female sample (left).

**Figure 2:**
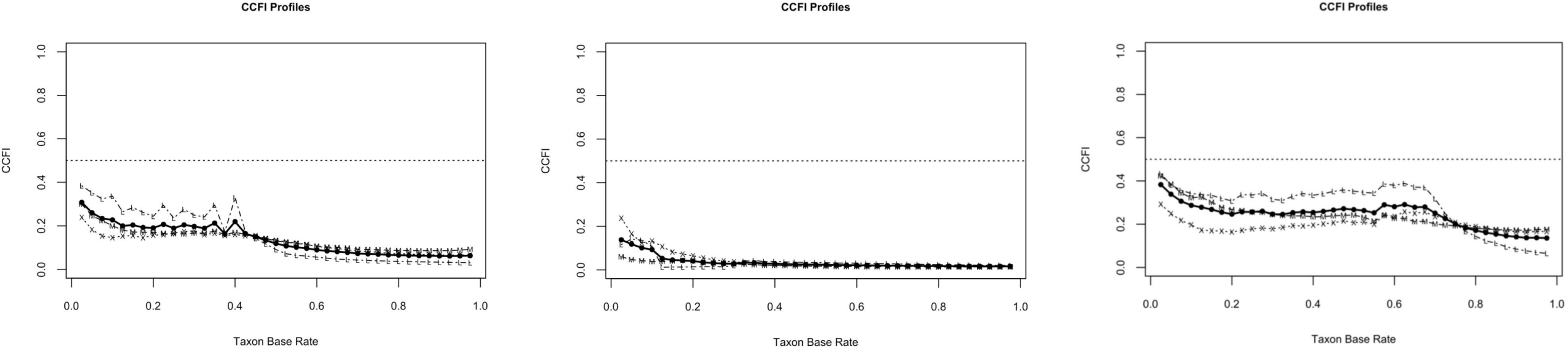
Results of the CCFI-profile analyses based on the ICD-11 indicator-set for the total sample (right), male sample (middle), and female sample (left).

The results of the CCFI profile analyses are depicted in **Figure 1** (DSM-5) and **Figure 2** (ICD-11). In each figure, separate curves are shown for the total sample, as well as for the male and female subgroups. The x-axis represents different assumed taxon base rates (i.e., hypothetical prevalence rates of IGD if it were categorical), and the y-axis shows the corresponding CCFI values. The horizontal dashed lines at 0.50 demarcate thresholds for interpreting the curve as dimensional, or taxonic. Based on both diagnostic frameworks, the CCFI curves consistently remain well below 0.50 across the full range of base rates (including the 5–50% range, which reflects plausible epidemiological values; see 24,27,34), indicating strong and stable support for a dimensional structure. This finding is particularly robust in the male subsample, where CCFI values approach 0.05 in the ICD-11 model—an exceptionally strong indication of dimensionality. While female subsamples show slightly higher CCFI values, these still remain below the 0.50 threshold throughout, suggesting no meaningful taxonic pattern. Summing up, for the DSM-5-based analysis (**Figure 1**), the mean CCFI value for the total sample was 0.311, clearly below the threshold of 0.45. Stratified analyses yielded CCFI values of 0.162 for males and 0.390 for females, indicating robust support for dimensionality in both subgroups, albeit with slightly higher values among females. Similarly, for the ICD-11-based indicator set (**Figure 2**), dimensionality was again supported with a total sample CCFI of 0.175, and subgroup-specific values of 0.046 for males and 0.268 for females. This pattern suggests that the dimensional structure of IGD symptoms is not only evident in the DSM-5 framework, but also persists when applying the ICD-11 criteria, thereby strengthening the generalizability and diagnostic relevance of the findings. These findings suggest that IGD symptoms among adolescents are better represented as existing along a continuum of severity, rather than reflecting a discrete diagnostic category. Importantly, this pattern of results was observed consistently across both diagnostic frameworks, supporting a dimensional structure not only for the DSM-5-based criteria but also for the ICD-11 conceptualization of Gaming Disorder.

## Discussion

The present study evaluated the latent nature of IGD-symptoms using data obtained from four large samples of German ninth graders. While earlier analyses have addressed the prevalence of IGD in this cohort (2013; see 27), the present study makes a novel contribution by investigating whether IGD is best conceptualized as a categorical or dimensional construct using taxometric methods. To the best of our knowledge, no previous study has applied such methods to examine the latent structure of IGD. Results strongly suggested a dimensional solution, which at least in part, does not correspond to taxometric studies of PG (21–23). A dimensional structure of IGD would have important theoretical as well as clinical implications: First, as highlighted by Meehl (13), dimensional structures typically reflect the additive and interactive effects of numerous risk factors, rather than a discrete etiological boundary. This perspective aligns with contemporary psychopathology research, suggesting that complex mental health conditions such as IGD emerge from distributed, cumulative processes rather than from clearly demarcated categories. In this regard a dimensional model of IDG symptoms enable the systematic inclusion of subclinical individuals and populations in the research context. This not only improves the ecological validity and generalizability of findings but also resonates with network-based models of psychopathology, in which symptoms are conceptualized as interconnected and mutually reinforcing elements rather than mere indicators of an underlying latent disorder (39). Second, existing screening and diagnostic tools - such as the Computer Game Addiction Scale (CSAS), the Internet Gaming Disorder Scale – Short Form (IGDS9-SF), and the Gaming Disorder Test (GDT) - should be reevaluated to ensure they capture gradations of severity, rather than merely discriminating between (not existent) latent groups. Items must be sensitive across the full range of symptom expression - especially in adolescents, where early symptoms may not meet clinical thresholds but nonetheless indicate elevated risk (40) Third, the conventional separation between prevention and intervention becomes increasingly arbitrary in a dimensional framework. Mild symptoms might already warrant low-intensity interventions, and preventive strategies should not be limited to those without current diagnosis. Instead, prevention and treatment can be conceptualized as points on a continuum of therapeutic intensity, tailored to symptom severity rather than diagnostic category. In this context in seems tenable to define treatment success (as measured by overall symptom strain) as an external criterion to develop multiple diagnostic thresholds regarding IGD Dimension i.e. which degree of symptom strain warrants what degree of treatment. Furthermore, Future prevention/intervention studies should critically reevaluate the typical diagnosis based ex-/inclusion criteria. Forth, beyond psychometric and clinical considerations, the latent structure of Internet Gaming Disorder (IGD) also carries implications for how individuals are labeled and perceived within clinical, scientific, and public discourse. As highlighted in previous research (41), the way a psychological construct is communicated - categorically or dimensionally—can substantially influence public and professional perceptions. Specifically, framing a construct as taxonic, for instance by labeling individuals as having or not having a “disorder,” tends to evoke the notion of a stable, binary distinction and may inadvertently contribute to stigmatizing those affected. In contrast, describing symptoms as dimensional supports the understanding that problematic behaviors exist on a continuum, are potentially transient, and may be modifiable through intervention. In the case of IGD, the dimensional structure observed in our data supports a more nuanced communication of gaming-related problems. Rather than labeling adolescents as disordered once they cross a diagnostic threshold, it may be more appropriate - and ethically responsible - to describe them as exhibiting elevated levels of problematic gaming behavior. This approach acknowledges the severity of their symptoms without implying permanence or identity-defining pathology. It may also help reduce self-stigmatization and social stigma, which are known barriers to treatment engagement, especially among youth. Furthermore, adopting a dimensional communication style aligns with the broader principles of person-centered care and mental health destigmatization. It allows practitioners to convey that symptom levels can change over time, encourages individuals to seek support earlier, and enables relatives to respond with greater understanding and empathy.

### Limitations

There are many strengths of this study, including the very large and representative sample. However, the study has some limitations. First, self-reports were the only data source used, so it is possible that the results exhibit monomethod bias (40, 42). When attempting to replicate our findings in future studies, investigators should ensure that other data sources are used, such as other self-report-measures, teacher/parent reports, clinical interviews, and/or observational measures. A multi-informant approach may improve diagnostic precision and enhance construct validity. Parent-reports have been shown to offer incremental validity in identifying problematic gaming, especially in younger adolescents (43). Likewise, teacher assessments could contribute relevant information on academic impairment or social withdrawal-symptom domains highly relevant for IGD but often underreported in self-assessment. Incorporating such perspectives would allow for a more comprehensive and ecologically valid evaluation of symptom severity and functional impairment. Second, an important limitation concerns the assessment of ICD-11 criteria. While the CSAS captures the core features of IGD as outlined in both DSM-5 and ICD-11, it was not specifically developed for the ICD-11 framework (in contrast to, e.g., the Gaming Disorder Test; 32). Thus, our ICD-11-based findings should be interpreted with caution regarding their diagnostic precision and replicability, especially in cross-study comparisons. Future studies should consider the direct operationalization of ICD-11-specific criteria to enable a more precise alignment. For the present study, however, the CSAS provides a conceptually and psychometrically adequate approximation of both diagnostic systems. Third, data presented here is limited to the age group of ninth graders with a mean age of 15 years. However, IGD is common among adolescents and might express differently in older age groups, which is especially assumed for more severe symptoms as for example the risk/loose criterion (27). In addition, longitudinal data suggest that IGD symptoms in adolescents are often transitory. A recent meta-analysis found that only around 33–38 % of adolescents who met criteria for IGD at one point continued to meet diagnostic thresholds two years later (44). Longitudinal and cross-sectional studies in broader age ranges are needed to clarify potential age- or context-dependent variations in the latent structure of IGD. Forth, the generalizability of the findings to clinical populations remains uncertain. Individuals seeking treatment for disordered gaming often present with increased symptom severity and frequently report comorbid psychiatric conditions, particularly depression (45). Importantly, IGD and depression share several symptomatic features, including anhedonia, social withdrawal, academic or occupational decline, fatigue, and disturbances in circadian rhythms (46). These overlapping characteristics may influence how individuals interpret and respond to diagnostic items, thereby affecting indicator performance in taxometric analyses. It therefore remains unclear whether the dimensional structure identified in the present community sample would replicate in clinical contexts, where symptom profiles and latent constructs may differ. Fifth, since the present analyses were based on a German sample, it would be valuable to examine the latent status of IGD symptoms in adolescent samples from other cultural contexts, such as Asia, Latin America, or the United States. This appears particularly important considering recent findings indicating substantial cross-cultural variation in IGD prevalence rates among adolescents. For instance, prevalence rates of up to 11.7% have been reported in China, 19.3% in South Korea, and 12.3% in Brazil, whereas markedly lower rates are observed in the United States (3.3%) and across European countries, ranging from 1.2% in Norway to 9.6% in Spain (47). Such differences cannot be attributed solely to infrastructural, educational, or public health factors, nor to regulatory conditions. They are also likely shaped by cultural variables that influence both collective and individual understandings of health, as well as the social responses to problematic media use in families, peer groups, schools, or workplaces. Against this background, it seems particularly worthwhile to replicate the present taxometric analyses -conducted in a horizontal-individualistic cultural context [autonomy combined with a strong emphasis on equality; see (48)] - in societies characterized by different cultural orientations (e.g., Asia, North, or Latin America). Such cross-cultural replications could help determine whether there is a universal latent structure of IGD symptoms or whether it varies across cultural contexts. At least one study has provided initial evidence of differences in measurement models between individualistic cultures (i.e., United States and Australia) and collectivistic cultures (i.e., Turkey and Sri Lanka), suggesting possible cultural divergence in the internal structure and interpretation of the IGD construct [see (49)]. Sixth, although the dataset meets several established criteria for the application of taxometric procedures, the within-group correlations among indicators slightly exceed the commonly recommended threshold of *r* <.30, which is typically expected under categorical latent structures. According to Ruscio et al. (19), such difficulties in constructing indicators with sufficiently low intercorrelations may themselves be indicative of a dimensional rather than categorical latent structure, as high within-group correlations are less problematic—and even expected—under dimensional models.

## Conclusion

Decisions in clinical practice regularly remain categorical in nature (treatment vs. no treatment). In order to empirically validate such a categorical decision process in the future, it seems necessary to define diagnostic thresholds regarding IGD-symptom burden based on external criteria (19, 50). Related to IGD, such external criteria could represent general functioning level (e.g., IGD-related incapacity to work or truancy), or suicidal ideation or suicide attempts.

While the dimensional structure of IGD observed in this sample may provide valuable insight into symptom expression among adolescents in general education settings, caution is warranted when extending these findings to other populations. In particular, it remains an open question whether similar latent structures would emerge in adult populations, in clinical samples with elevated symptom severity, or in at-risk groups such as youth with comorbid psychopathology or problematic digital media use. Future research is needed to determine whether the dimensional pattern holds across these distinct subgroups and to examine potential differences in symptom thresholds, course, and functional impairment.

## Data Availability

The raw data supporting the conclusions of this article will be made available by the authors, without undue reservation.
